# First-in-Human Percutaneous Use of a Cerebral Flow-Diverting Stent in a Large Coronary Aneurysm

**DOI:** 10.1016/j.jscai.2023.101232

**Published:** 2024-03-06

**Authors:** Chintan P. Patel, Alexander L. Coon

**Affiliations:** a Interventional Cardiology—Atria Heart in Collaboration with Honor Health, Scottsdale, Arizona; b Department of Neurosciences, Carondelet Neurological Institute, St. Joseph’s Hospital, Tucson, Arizona

**Keywords:** coronary aneurysm, flow-diverting stent, percutaneous treatment of coronary aneurysm

## Abstract

A 58-year-old man presenting with angina was found to have a large coronary aneurysm on angiography. After coronary bypass and multiple ST-elevation myocardial infarctions over the following months, the decision was made to exclude the aneurysm with a flow-diverting stent, which reduced flow to the aneurysm and left the patient asymptomatic since the procedure. This is the first reported use of a cerebral flow-diverting stent for treatment of a coronary aneurysm.

The prevalence of coronary artery aneurysms has been reported to be 0.02% to 0.2%.^1^^,^^2^ The majority are asymptomatic; however, they can lead to ischemia, thrombosis, fistula, or possible rupture. There are no randomized studies to evaluate the most effective therapy for these patients. Covered stenting, coil embolization, and surgical bypass with exclusion of these aneurysms have been reported. Flow-diverting stents (FDS) are routinely used in the treatment of saccular and fusiform cerebral aneurysms (Figure 1). FDS have demonstrated occlusion rates near 80% in cerebral aneurysms.^3^ In peripheral and visceral arterial aneurysms, FDS have demonstrated a total occlusion rate of approximately 90% with a stent patency rate of 88%.^4^

**Figure 5:**
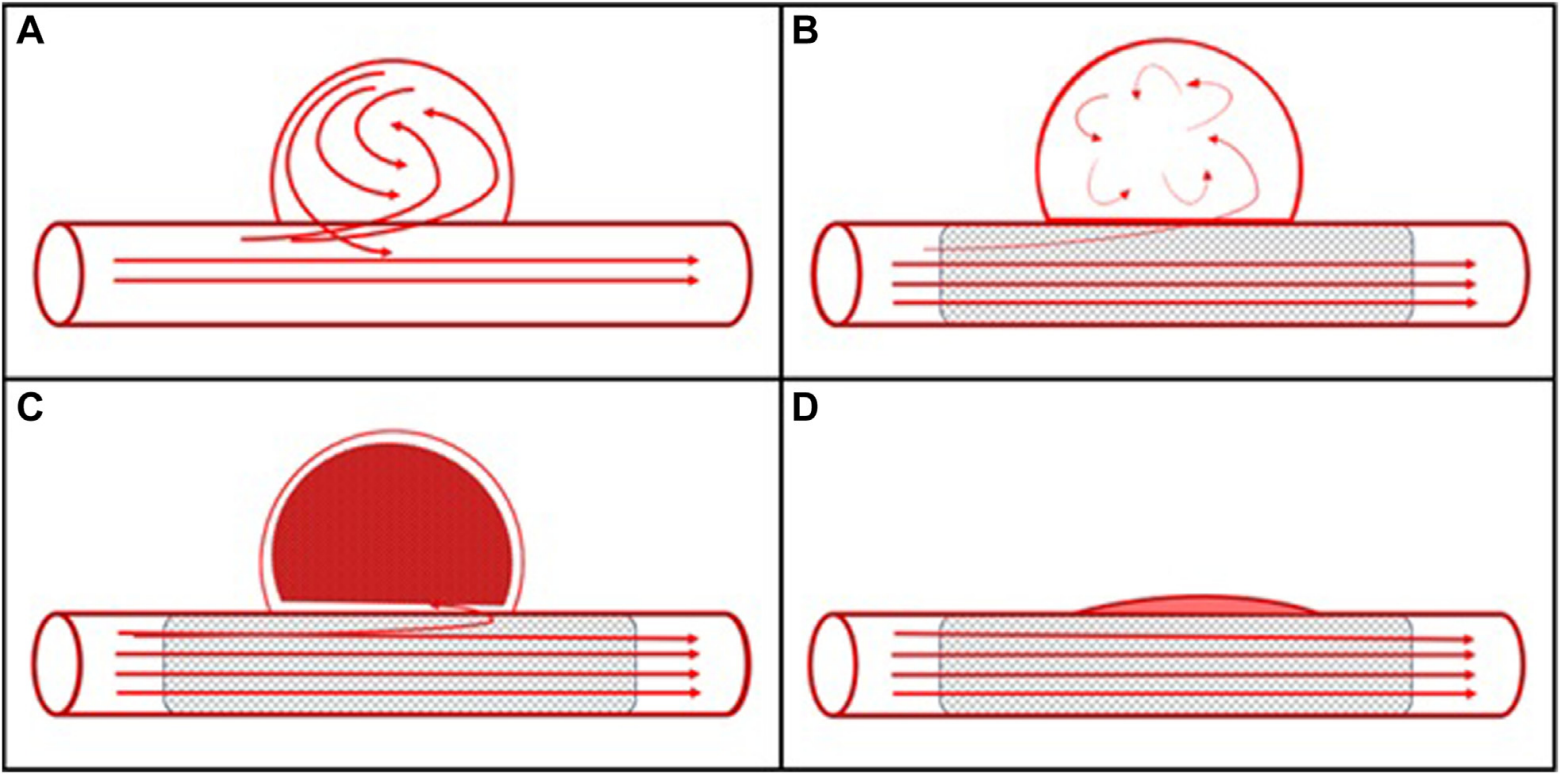
**Flow-diverting stent mechanism.** (**A**) Flow turbulence in the aneurysmal segment as it relates to the laminar flow down the native vessel. (**B**) Placement of a porous stent across the aneurysm continues to allow for laminar flow but starts to disrupt flow within the aneurysm. (**C**) Aneurysmal thrombosis starts to occur due to stasis and further minimizes flow over hours to days. (**D**) Over months, total thrombosis and vessel remodeling with reabsorption of the aneurysm and neointimal coverage occurs.

We present a novel percutaneous solution for a large coronary aneurysm causing multiple adverse coronary events. To the best of our knowledge, this is the first use of an FDS in a coronary artery.

A 58-year-old man presenting with typical anginal symptoms had a diagnostic coronary angiogram showing a large fusi-saccular aneurysm exceeding 10 mm in diameter and 40 mm in length in the midleft circumflex (LCX) ending just prior to a bifurcation of the dominant left posterior descending artery (LPDA) and a medium-caliber second obtuse marginal (OM2) (Figure 2). Both the LPDA and OM2 have calcified 80% lesions. Initially, he was sent for coronary artery bypass grafting; however, 3 months later, he presented with an inferior ST-elevation myocardial infarction (STEMI) with TIMI 1 flow in the LPDA and OM2. This was treated medically with rivaroxaban 20 mg daily in addition to dual antiplatelet therapy. Three weeks later, he had another STEMI in the same territory while compliant with rivaroxaban and dual antiplatelet therapy. This time he was treated with a balloon angioplasty of the ostial LPDA with residual 70% stenosis. The patient was kept in-house on heparin. Given that the aneurysm was the culprit and showering thrombus distally, leading to STEMI, the decision was made to exclude the aneurysm. The patient was brought back to the laboratory 5 days later. Using right radial access, coronary lithotripsy from the distal LCX into ostial LPDA was performed with placement of a 4-mm × 48-mm Synergy drug-eluting stent (Boston Scientific) from LPDA back to the mid-LCX across the aneurysm. This served as a scaffold, spanning the aneurysm. We then deployed a 4.5-mm × 40-mm Surpass Evolve 64-wire braided cobalt-chromium FDS (Stryker Neurovascular) through the scaffold (Figure 3). Immediately after placement, there was disruption of flow into the aneurysm. On repeat angiography 7 weeks later (Supplemental Video 1; Figure 4), there was interval remodeling with a vast improvement in laminar flow through the stented portion, with only trace residual flow into the aneurysm neck. The FDS promoted laminar flow through the natural vessel architecture while decreasing flow into the aneurysm itself, thus promoting thrombosis of the aneurysm (Figure 5). The patient has remained asymptomatic and is completing his cardiac rehabilitation.

**Figure 1:**
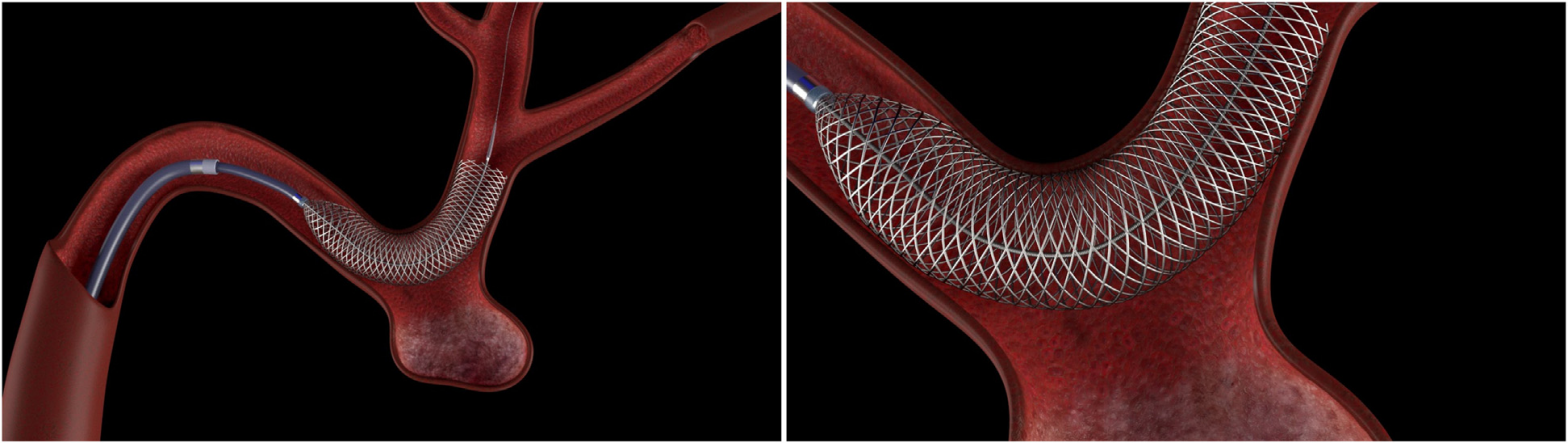
**Flow-diverting stent modelwith aneurysm.** Left: Flow-divertying stent model with aneurysm. Right: Detailed view. Source: Purchased stock photo from www.turbosquid.com.

**Figure 2:**
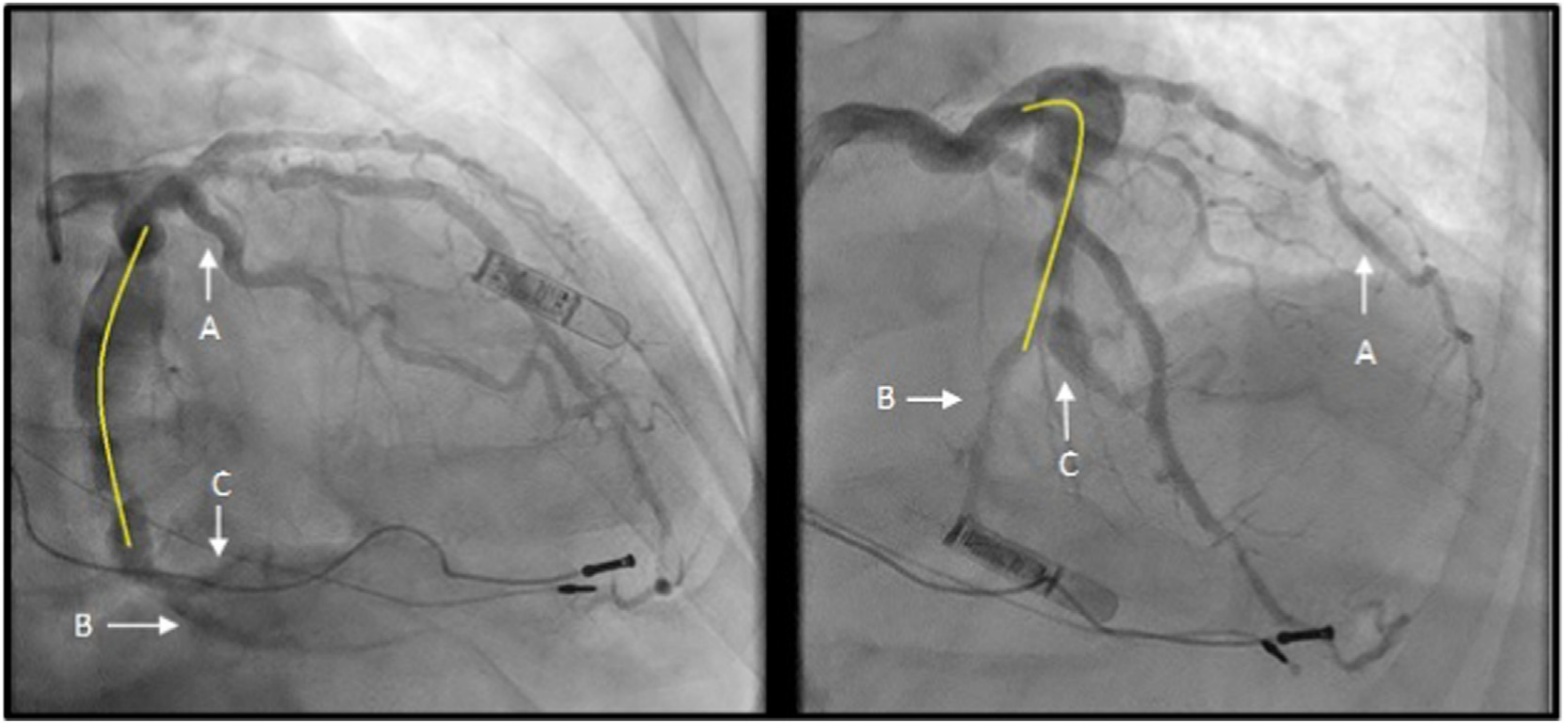
**Initial diagnostic coronary angiogram.** A large fusi-saccular aneurysm in the midleft circumflex with area to be covered with the flow-diverting stent (yellow). The (**A**) first obtuse marginal was the only midleft circumflex branch bypassed during the initial surgical procedure, with (**B**) the left posterior descending artery and (**C**) second obtuse marginal receiving native flow through the aneurysm.

**Figure 3:**
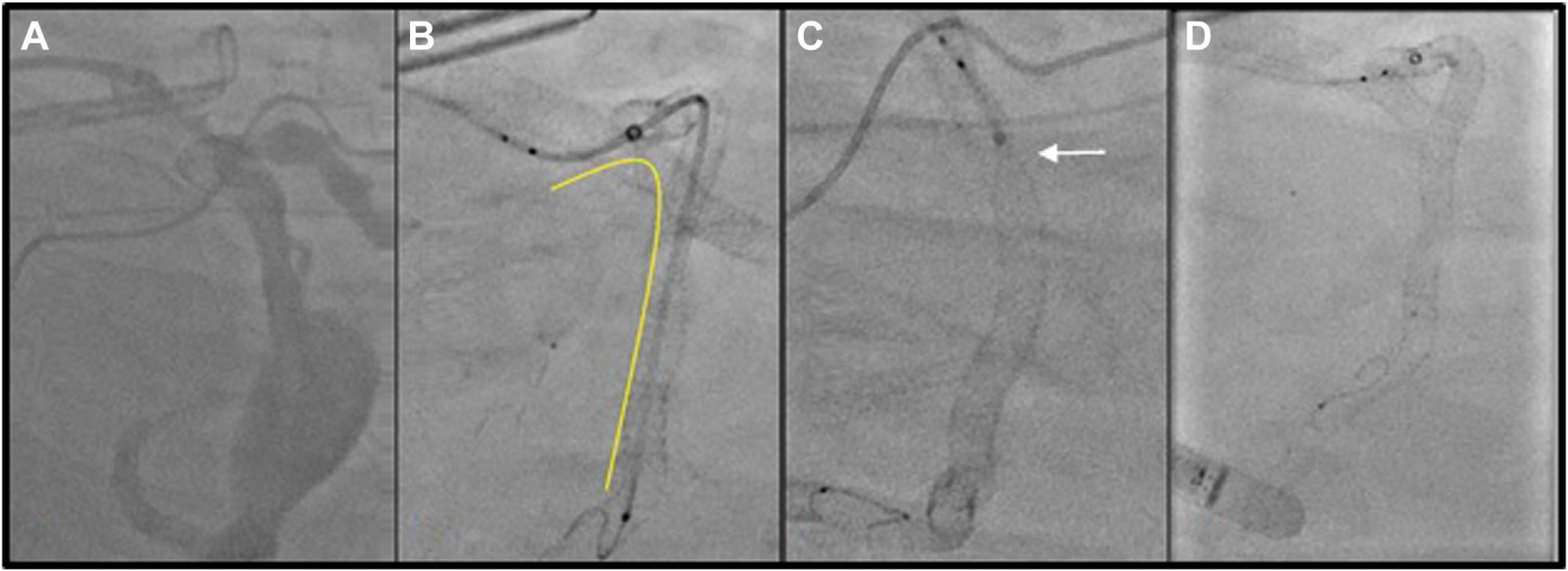
**Deployment of the Stryker Surpass Evolve flow-diverting stent (FDS).** (**A**) Angiogram after placement of Synergy stents from the left posterior descending artery through the aneurysm and into the mid-LCX (scaffold). (**B**) Placement of the Surpass Evolve FDS delivery microcatheter (yellow). (**C**) Unsheathing self-expanding FDS (arrow). (**D**) Final deployment of the FDS.

**Figure 4:**
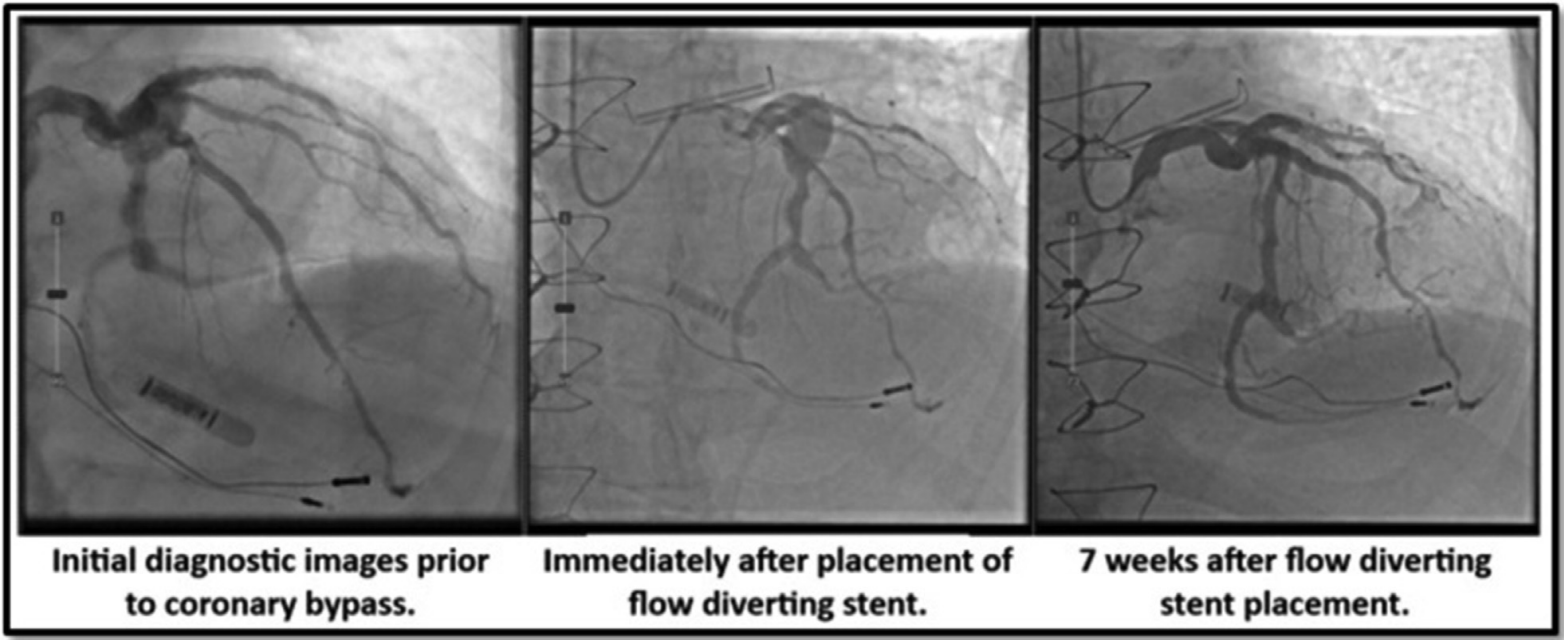
**Before and after flow-diverting stent placement with follow-up.** Seven-week follow-up angiogram showing near-complete resolution of the aneurysm with preservation of left posterior descending artery and second obtuse marginal patency.

## Supplementary material

Video 1 **Before and after flow-diverting stent placement with follow-up.** Seven-week follow-up angiogram showing near-complete resolution of the aneurysm with preservation of left posterior descending artery and second obtuse marginal patency.
